# Bullous pemphigoid following COVID-19 vaccine: An autoimmune disorder

**DOI:** 10.1016/j.amsu.2022.104266

**Published:** 2022-07-31

**Authors:** Aashish, Aneesh Rai, Govinda Khatri, Priya, Mohammad Mehedi Hasan

**Affiliations:** a Dow University of Health Sciences, Karachi, Pakistan; b Department of Biochemistry and Molecular Biology, Faculty of Life Science, Mawlana Bhashani Science and Technology University, Tangail, Bangladesh

Bullous pemphigoid (BP) is an autoimmune blistering dermatologic disease that is clinically differentiated by tense bullae that can grow on normal or erythematous skin. A subepidermal blister with varying degrees of dermal inflammation is the most prominent histologic characteristic [1]. Bullous Pemphigoid is characterized by IgG autoantibodies directed against self-antigens at the basement membrane zone; these antigens are BP180 and BP230. Both of these antigens are essential components of the hemidesmosome, that are responsible for epidermal-dermal adhesion [2]. BP230 is a plakin family protein that is an intracellular component of the hemidesmosome, whereas BP180 is a transmembrane glycoprotein with an extracellular domain - NC16A - that is the major antigenic epitope in BP [2].

Between December 24 and April 25, 2021, the American Academy of Dermatology/International League of Dermatological Societies COVID-19 Dermatology registry, recorded 733 cases of skin responses reported following COVID-19 vaccination [3]. Recent research indicates that BP-like symptoms have been observed after immunization with a range of vaccines, including measles, varicella-zoster, influenza, hepatitis B, and human papillomavirus vaccines [4,5]. According to one study conducted by Tomayko MM et al. patients who develop bullous pemphigoid after 1st vaccine should receive the 2nd dose as well, despite the autoimmune reaction following 1st dose [6]. The diagnosis of bullous pemphigoid can be confirmed by skin biopsy including both direct and indirect positive serum immunofluorescence [7]. Skin Biopsy shows a subepidermal detachment along with an eosinophilic inflammatory cell infiltrating the superficial dermis.

Its etiopathogenesis is determined by genetic predisposition and specific triggering events. The relation between BP and basement membrane disruptions, such as trauma or burns; specific medicines, such as oral antidiabetics; or neurological conditions, such as Parkinson's disease or dementia, is well established. The latter relationship is explained in part by an autoimmune cross-reaction between the BP230 protein isoforms found in both the skin and the central nervous system [8,9]. Skin reactions following SARS-CoV-2 vaccination have been recorded in increasing numbers. According to recent research, there are two major groups of cutaneous symptoms produced by vaccination, which we both noticed in our patients: flares of pre-existing dermatoses as well as new reactions [10]. Autoimmune bullous responses to COVID-19 vaccinations are uncommon, with just a few cases recorded to date following the first or second dosage. Scars following the administration of COVID-19 vaccinations have previously been reported; however, those primarily comprise drug-induced hypersensitivity responses and, less frequently, DRESS syndrome or Stevens-Johnson syndrome [11].

The timing of these SCARs appears to correspond to that observed in those of BP patients, with a higher tendency for the development of a response during the first 24 h after treatment. However, even several days following dosing, new-onset cutaneous signs have been reported. These flare-ups are potentially treatable and preventive with precautionary measures such as previous screening for higher-risk individuals with particular human leukocyte antigen (HLA) types and autoimmune disorders, as well as involvement in vaccination trials. Protection of these populations against SARS-CoV-2 remains a priority, particularly given the emergence of new variations and rising incidence [12]. Several of these vaccine reactions were found among the pediatric population.

The binding of *anti*-NC16A autoantibodies to BP180 activates several pathways, which include activation of complement, chemotaxis of neutrophils with the release of proteases and elastases, and blister formation [13]. The predominant *anti*-NC16A IgG subclasses are IgG1 and IgG3 followed by IgG4. Both IgG1 and IgG3 bind to the basement membrane zone (BMZ) and fix complement whereas incubation with IgG4 at the same time causes its deposition in the BMZ and blocks IgG1 binding which will prevent complement fixing, chemotaxis of neutrophils, and blister formation [14]. Autoantibody binding activates complement, recruits inflammatory cells, and releases proteolytic enzymes, triggering an inflammatory cascade that could be activated even by Th17 cell activation [15]. IgE autoantibodies, in addition to IgG, play a role in disease pathogenesis and can be detected by immunofluorescence studies and ELISA analyses in the skin and serum of BP patients [16]. A major histologic finding in BP is eosinophilic infiltration. In the murine model, eosinophils are essential for IgE-mediated blister formation in BP, acting as a cellular link between IgE autoantibodies and skin lesions [17].

The mechanism by which the COVID-19 vaccine cause Bullous Pemphigoid is still unknown. However, studies suggest that molecular mimicry may be linked to autoimmune responses following SARS-CoV-2 infection [18]. In genetically susceptible individuals, vaccination, on the other hand, may stimulate B and T-cell immunity, generating an autoimmune reaction. Furthermore, vaccination-induced inflammation may produce a disruption of the basement membrane, resulting in the generation of anti-basement membrane antibodies [19]. However, further research is needed to investigate the exact mechanism of COVID-19 vaccine-induced BP. The goal of BP treatment is to stop the progression of new lesions, relieve the itching and promote epidermal healing. The treatment options are determined by the severity of the disease as well as general health problems and comorbidities.

Topical corticosteroid creams that are directly rubbed on affected areas are used to treat mild cases of BP. For severe cases, oral corticosteroid like prednisone is used along with steroid-sparing drugs such as dapsone, azathioprine, or mycophenolate mofetil. Rituximab or IVIG infusions are used to treat refractory cases of BP [20]. Apart from medical therapy, BP can be controlled by caring for wounds, avoiding sunlight, and dressing comfortably.

## Ethical approval

NA.

## Sources of funding

NA.

## Author contribution

**Aashish:** Conceptualization, Writing – original draft. **Aneesh Rai:** Writing – original draft. **Govinda Khatri:** Writing – original draft. **Priya:** Writing – original draft. **Mohammad Mehedi Hasan:** Conceptualization, Writing - review & editing.

## Registration of research studies

1. Name of the registry: NA
2. Unique Identifying number or registration ID: NA
3. Hyperlink to your specific registration (must be publicly accessible and will be checked): NA

## Consent

NA.

## Guarantor

Mohammad Mehedi Hasan.

Department of Biochemistry and Molecular Biology, Faculty of Life Science, Mawlana Bhashani Science and Technology University, Tangail, 1902, Bangladesh. Email: mehedi.bmb.mbstu@gmail.com.

## Declaration of competing interest

NA.
